# Real-World COVID-19 Vaccine Protection Rates against Infection in the Delta and Omicron Eras

**DOI:** 10.34133/research.0099

**Published:** 2023-04-05

**Authors:** Yuru Zhu, Jia Gu, Yumou Qiu, Song Xi Chen

**Affiliations:** ^1^ Center for Statistical Science, Peking University, Beijing, China.; ^2^ Department of Statistics, Iowa State University, Ames, IA, USA.; ^3^ School of Mathematical Science and Guanghua School of Management, Peking University, Beijing, China.; ^4^ Pazhou Lab, Guangzhou, China.

## Abstract

The real-world vaccine protection rates (VPRs) against the severe acute respiratory syndrome coronavirus 2 (SARS‑CoV‑2) infection are critical in formulating future vaccination strategies against the virus. Based on a varying coefficient stochastic epidemic model, we obtain 7 countries’ real-world VPRs using daily epidemiological and vaccination data, and find that the VPRs improved with more vaccine doses. The average VPR of the full vaccination was 82% (SE: 4%) and 61% (SE: 3%) in the pre-Delta and Delta-dominated periods, respectively. The Omicron variant reduced the average VPR of the full vaccination to 39% (SE: 2%). However, the booster dose restored the VPR to 63% (SE: 1%) which was significantly above the 50% threshold in the Omicron-dominated period. Scenario analyses show that the existing vaccination strategies have significantly delayed and reduced the timing and the magnitude of the infection peaks, respectively, and doubling the existing booster coverage would lead to 29% fewer confirmed cases and 17% fewer deaths in the 7 countries compared to the outcomes at the existing booster taking rates. These call for higher full vaccine and booster coverage for all countries.

## Introduction

Severe acute respiratory syndrome coronavirus 2 (SARS‑CoV‑2) has been circulating globally with a sequence of emerging variants since the start of the pandemic. Particularly, the Delta and Omicron variants have contributed to surges in the infected cases across the globe due to their high transmissibility [1,2]. To prevent the spread of coronavirus disease 2019 (COVID-19), vaccines have been rolled out since late 2020, while the booster shots were started since June 2021. Clinical trials or observational studies have been made to evaluate the effects of a vaccine or an arrangement of mixed vaccines [3–9]. It is found that the vaccine efficacies of the 2-dose vaccination against the original SARS-CoV-2 strain ranged from 50.7% to 95% [3,4,10], but waned against Delta and Omicron variants. The vaccine effectiveness ranged from 82.8% to 94.5% against Delta and from 48.9% to 75.1% against Omicron for 2 doses of Pfizer, AstraZeneca, or Moderna vaccines [8]. The vaccine efficacy is defined as one minus the relative risk in the randomized controlled clinical trials [11], and the vaccine effectiveness is valued in observational studies, which is one minus the hazard ratio in cohort studies [6] and one minus the odds ratio in case–control studies [5]. Observational studies were more common in the Omicron era.

The booster dose had been shown to increase protection against infection. For homologous or heterologous booster doses of Pfizer, AstraZeneca, and Moderna, the effectiveness was 82.3%–97.0% against Delta and 55.6%–73.9% against Omicron, with higher effectiveness using Moderna as the heterologous booster dose [8]. The vaccine effectiveness was 51.0% for 3 doses of Sinovac against Omicron [12], which increased to 63.6% by Sinovac as primary with one Pifzer booster [13]. See Table S1 for the detailed vaccine efficacy and effectiveness discovered by the existing clinical and observational studies. However, the real-world performance of vaccines at the country’s population level that we call the vaccine protection rate (VPR) is largely unknown.

Different from vaccine efficacy and effectiveness, the real-world VPR is defined as one minus the ratio of the infection rate of the vaccinated over the infection rate of the unvaccinated population of a country. The VPR measures the combined effectiveness of vaccines administrated in a country at a particular age distribution and nonpharmaceutical intervention (NPI) measures against COVID-19. The impacts of these factors are not necessarily evaluated in the homogeneous clinical trials, cohort studies, or case–control studies. Indeed, the conventional vaccine efficacies are pegged to a specific vaccine or a mix of vaccines in the clinical trials after excluding a certain part of the population, which may not conform to the population characteristics of the country. Therefore, the available vaccine efficacy or effectiveness does not necessarily reflect the vaccine immunity level of the whole population against different variants of the SARS-CoV-2 virus. Hence, it is crucial to obtain the real-world VPRs of a country to timely evaluate the effect of receiving the full and the booster vaccination on protecting the population in the whole country, which can provide quantitative evidence for the effectiveness of booster vaccination, dispel public doubts about the necessity of booster vaccination, and contribute to the public health policy decision-making.

Various models were proposed to study the spread of COVID-19, the behavior of SARS-CoV-2 cells’ infection, and some related biological mechanisms, such as the fractional order models [14,15], the deterministic compartment models [16,17], and the stochastic epidemic models [18–20]. Using the daily epidemiological and vaccination data, which include the cumulative numbers of confirmed cases, deaths, recoveries, and people having received the partial, full, and booster vaccination, we construct a varying coefficient stochastic epidemic model with 11 compartments (flow diagram in Fig. 4) and develop an estimation procedure for the real-world VPRs as well as the key parameters quantifying the dynamic infection, death, and recovery rates, which comprehensively reflect the COVID-19 dynamics and NPIs. Compared with existing studies on the effect of vaccination [16,17], we do not assume permanent and full immunity of the vaccines and previous infection while incorporating the stochastic natures of the epidemics with time-varying infection rate due to varying levels of NPI and self-protective measures, and allow asymptomatic infection, infection before clinic confirmation, vaccine breakthrough, reinfection, and different levels of immunity induced by different vaccine doses. The nonparametric time-varying infection rate in our model is better suited for the COVID-19 pandemic as both the virus transmission rate and the NPI measures change over time.

We considered 7 countries that are representative for different types of vaccines with a sufficient number of confirmed cases (more than 10% of the total population) after vaccination. Specifically, the results of the US may be used to show the effect of mRNA vaccines (Pfizer and Moderna); the 3 European countries, UK, Italy, and Germany, mainly used a mixture of nonreplicating viral vector vaccines (AstraZeneca) and mRNA vaccines; the 2 south American countries, Brazil and Peru, utilized the inactivated vaccines (Sinopharm and Sinovac), AstraZeneca, and Pfizer; Turkey used the inactivated vaccines at the beginning and then started Pfizer. In this paper, the full dose means one dose for Janssen and 2 doses for the other brands to complete the primary vaccination. Those who have not completed the full vaccination are called partially vaccinated. The booster shot means one dose after full vaccination. The coverage rates of the partial, full, and booster vaccines in population are reported in Fig. 3A, which shows that 62.3%–79% of the population in the 7 countries have taken the full shots on 2022 March 15, but the coverage rates of booster shots were much lower, ranging between 29.1% in the US and 63.4% in Italy.

## Results

For each country, we estimate the VPRs in the 6 consecutive nonoverlapping post-vaccine periods: the pre-Delta, Intervening I, Delta-dominated, pre-Omicron, Intervening II, and Omicron-dominated periods. Details of these periods are provided in Materials and Methods.

### Vaccine protection rates

**Fig. 1.**
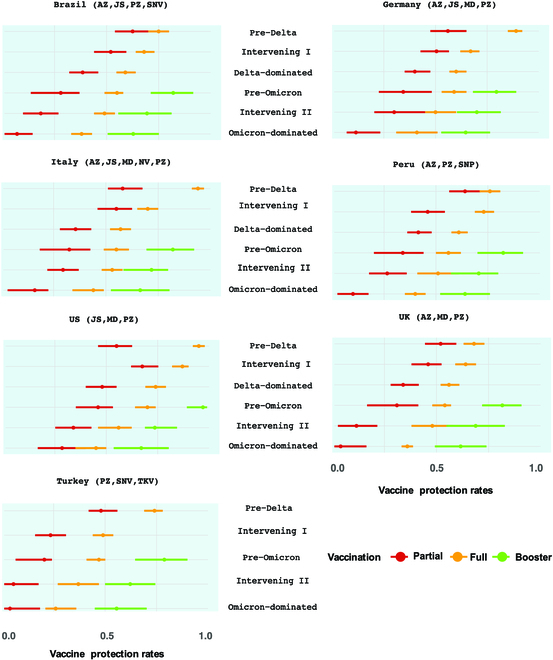
Estimated vaccine protection rates of partial, full, and booster vaccination in the 7 countries over the 6 periods with the 95% confidence interval bars. The vaccines used are reported in the parentheses (AZ, AstraZeneca; JS, Janssen; MD, Moderna; NV, Novavax; PZ, Pfizer; SNP, Sinopharm; SNV, Sinovac; TKV, Turkovac).

The real-world VPRs for the partial, full, and booster vaccination in the 7 countries in the 6 post-vaccine periods are reported in Fig. 1 with detailed numerical values in Table S3. It shows that before the booster vaccination, both the partial and full vaccination were largely protective against the COVID infection in the pre-Delta period with the VPRs in the 7 countries ranging from 48% to 64% and from 68% to 95% for the partial and full vaccination, respectively. However, the Delta variant had caused waning VPRs of the partial and full vaccination. Specifically, the average VPR of the partial vaccination decreased from 57% (SE: 2%) in the pre-Delta period to 40% (SE: 2%) in the Delta-dominated period, suggesting that only the partial shot was insufficient to protect against the Delta variant. Despite the fact that the Delta variant also reduced the average VPR of the full dose from 82% (SE: 4%) in the pre-Delta period to 61% (SE: 3%) in the Delta-dominated period, the average VPR of the full vaccination remained above the WHO recognized 50% level of vaccine efficacy in most countries except Turkey (Fig. 1). The coming of Omicron had reduced the VPRs of the partial and full vaccination in the 7 countries to less than 50%. In the Intervening II period, the VPRs were 5.5% to 34% (average: 22.2%, SE: 4.0%) for partial vaccination and 37% to 56% (average: 49.1%, SE: 2.3%) for full vaccination. When the Omicron variant became prevalent, VPRs were even lower, which were 3.8% to 28.5% (average: 11.5%, SE: 3.3%) and 26% to 45% (average: 38.6%, SE: 2.4%) for the partial and full vaccination, respectively.

The booster shot was started in the pre-Omicron period when the Delta was dominant. Our study shows that it readily restored the VPRs to 78.8% to 97% (average: 83.3%, SE: 2.3%), which means that the booster vaccination’s VPRs were 20.5% to 31.8% (average: 26.8%, SE: 1.2%) higher than those of the full vaccination against the Delta variant. In the Omicron-dominated period, the booster shot’s VPRs ranged from 55.6% to 67.0% (average: 63%, SE: 1.4%), largely staying above the 50% threshold. These suggest that the booster shot provided enhanced and effective protection against both the Delta and Omicron variants.

The VPR for a specific vaccine brand is similarly defined as the relative reduction in the infection rate of the population with that type of vaccine compared to the population without any vaccine protection in a country. It is noted that the information on the number of vaccine intakes for different brands of vaccines is unavailable in the national level statistics, which prevents us from directly measuring the VPR for a specific vaccine brand in a country. However, our approach is readily applicable, should the data information be available. In that case, we would conduct a regression analysis on the relationship between the estimated VPRs of each country and the intake proportions of different vaccine brands of this country in a certain time period.

### Impacts of full and booster vaccines

To further evaluate the protection of the COVID-19 vaccination, we investigate the impacts of the full and the booster vaccination on the size of the epidemics and deaths. Five vaccination scenarios were designed: (a) no vaccination at all; (b) receiving the partial but no full vaccination; (c) receiving the partial and full vaccination but no booster shots; receiving the booster shots only at half (d) and twice (e) of the actual daily booster coverage rates. The impacts of these scenarios were projected using the stochastic epidemic model with the estimated parameters for each country. See the specific designs of the scenario analysis (SA) in Materials and Methods.

**Fig. 2.**
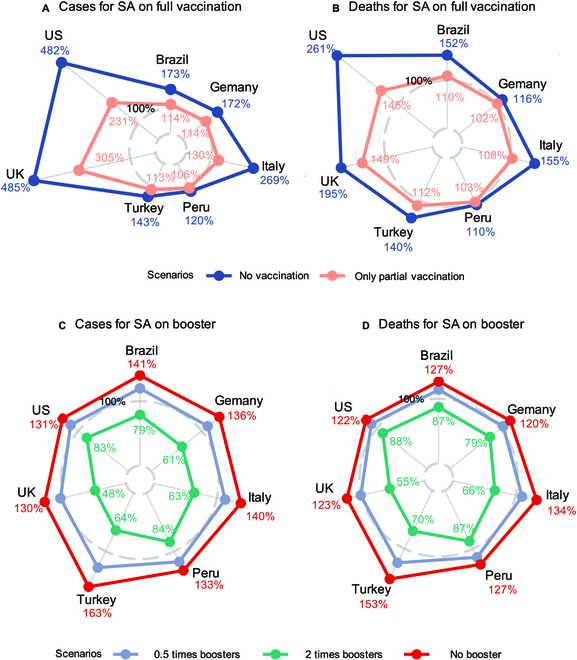
Radar plots on the proportions of the projected cumulative numbers of confirmed cases and deaths under 2 sets of scenario analyses (SA): the pre-booster vaccine periods from the start of vaccination to the start of the booster vaccination for each country (covering the pre-Delta, Intervening I, and Delta-dominated periods) under no and partial vaccination (A and B) and the post-booster periods from the start of the booster vaccination to 2022 March 15 for each country (covering the pre-Omicron, Intervening II, and Omicron-dominated periods) under the 3 scenarios with no booster, half the actual daily booster coverage rates (0.5 times boosters), and double the actual daily booster coverage rates (2 times boosters) (C and D), relative to their respective observed values in the 7 countries. The 100% gray dashed circles represent the observed situations.

The projected cumulative confirmed cases and deaths during the pre-booster vaccine periods from the start of vaccination to the start of boosters for the 7 countries (covering the pre-Delta, Intervening I, and Delta-dominated periods) under the no-vaccination (a) and only the partial vaccination (b) scenarios are shown in Fig. 2A and B with the detailed numerical values listed in Table S4. It shows that no vaccination at all would bring, respectively, 112,918 (CI: 96,142 to 129,695, percentage: 242%) and 825 (CI: 630 to 1,020, percentage: 83%) thousands increase in the cumulative confirmed cases and deaths in the 7 countries relative to the observed values under the actual vaccination arrangement. Under the only partial vaccination, the cumulative confirmed cases and deaths would increase by 39,697 (CI: 30,105 to 49,289, percentage: 85%) and 218 (CI: 124 to 312, percentage: 22%) thousands, respectively, in the 7 countries. These 2 scenario analyses show the significant benefit of the partial and the full vaccination.

**Fig. 3.**
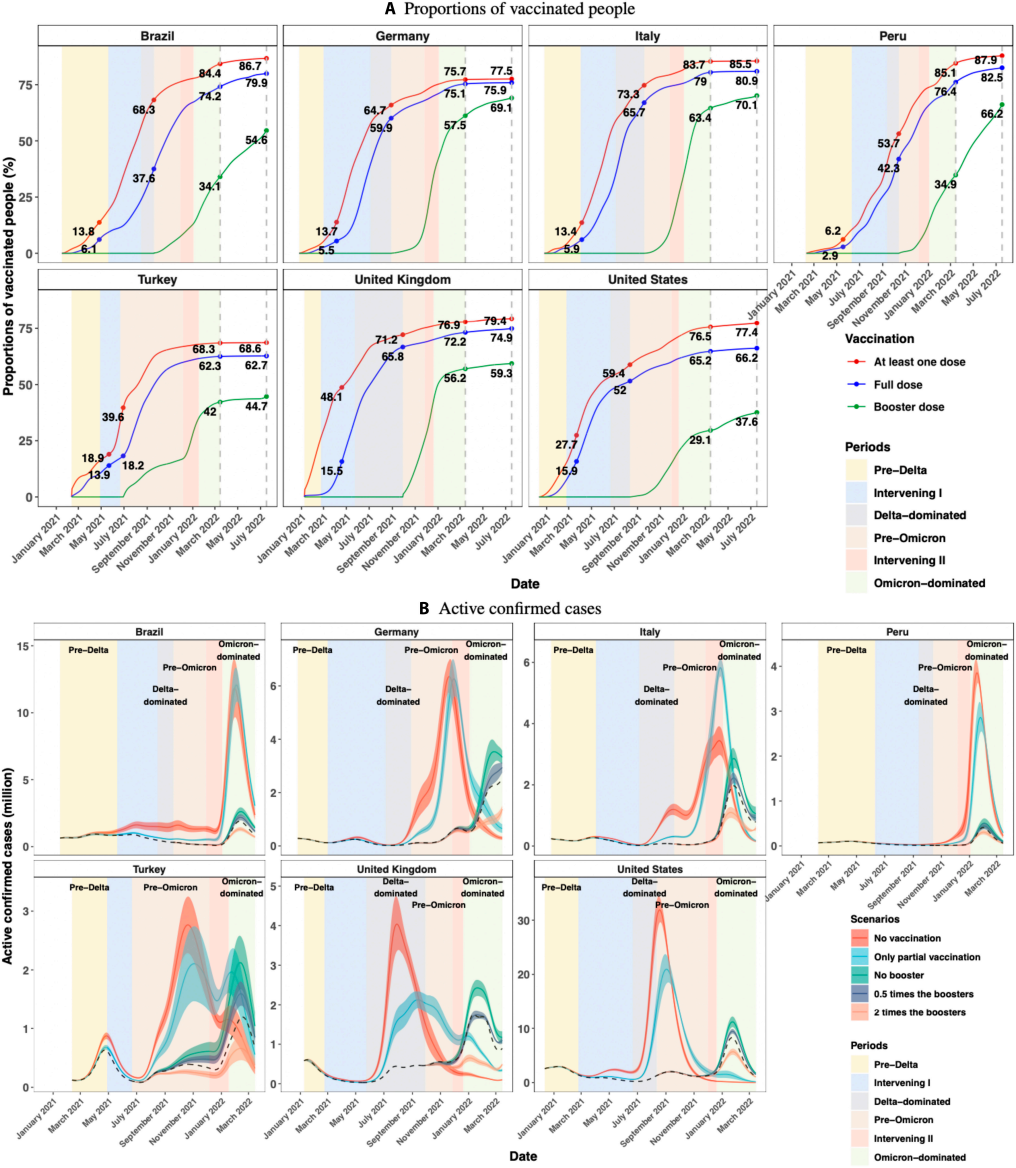
(A) Daily population proportions receiving at least one (red), full (blue), and booster (green) dose of vaccines. The dashed vertical lines mark 2022 March 15 and July 17. (B) The actual (black dashed lines) and the projected daily numbers (in millions) of active confirmed cases (color curves) and their 95% confidence bands (color area) under the 5 vaccination scenarios.

The lower increase in the confirmed cases and deaths under the no- and partial vaccination scenarios in Peru was due to its low and slow pace of vaccination, with only 6% and 3% of the population having received the partial and full vaccination within the first 100 days of vaccination. In contrast, 13.4% to 18.9% and 5.5% to 13.9% of the populations had been partially and fully vaccinated in Germany, Italy, Brazil, and Turkey, and the US and UK had the highest vaccination rates of 27.7% to 48.1% and 15.5% to 15.9% for the partial and full vaccination over the same period (Fig. 3A). That the US and UK had the highest vaccination rates in the first 100 days led to much higher numbers of cases and deaths under the no- and partial vaccination scenarios in Fig. 2A and B, as compared with the other countries.

Figures 2C and D display the projected cumulative confirmed cases and deaths during the post-booster periods from the start of booster shots to 2022 March 15 (covering the pre-Omicron, Intervening II, and Omicron-dominated periods) under the scenarios (c) to (e) regarding the booster vaccination, which kept the observed numbers of the partial and full doses as the baselines; see Table S5 for the detailed numerical values. It is shown that during the post-booster periods, not having the booster shots at all would mean 34,860 (CI: 23,543 to 46,177) and 143 (CI: 88 to 198) thousands more confirmed cases and deaths, respectively, in the 7 countries, amounting to 36% and 26% increases in the total confirmed cases and deaths, respectively. In the half-booster scenario, the increases in the confirmed cases and deaths would be less than those under the no-booster case, but still translate to 14,587 (CI: 8,024 to 21,150, percentage: 15%) and 66 (CI: 29 to 103, percentage: 12%) thousands more confirmed cases and death relative to the observed numbers, respectively, for the 7 countries in the post-booster period.

If the booster taking rates were doubled, we see decreases of 27,679 (CI: 21,234 to 34,124, percentage: 29%) and 94 (CI: 62 to 126, percentage: 17%) thousands in total confirmed cases and deaths for the 7 countries in the post-booster period. It is noted that the relatively large reductions in the confirmed cases and deaths in Germany, Italy, Turkey, and UK under the double-booster scenario were due to their actual higher (more than 40%) rates of taking the booster shots by 2022 March 15 (Fig. 3A). In contrast, the US, Brazil, and Peru had lower booster taking rates, which led to smaller amount of reductions in the confirmed cases and deaths as compared to the other countries.

To further evaluate the dynamics of the COVID-19 epidemic with respect to different vaccination strategies, we report in Fig. 3B the observed and the projected daily numbers of active confirmed cases (those confirmed infective people who have not recovered or died), to reflect the potential real-time demand on the hospital system under the 5 scenarios. It shows that, compared to the observed time series of the active confirmed cases, the peaks of the active confirmed cases would be much elevated and happen much earlier under the no- and partial vaccination scenarios. In particular, the numbers of active confirmed cases in Germany, Italy, Turkey, the UK, and the US would peak when the Delta was dominant. It also shows that the full and the booster shots significantly delayed the timing and flattened the magnitude of the infection peaks in the 7 countries, and in particular, protected the populations in the more lethal pre-Omicron era. As shown in Table S6, the projected numbers of active confirmed cases would exceed the observed peaks for 70 to 111, 65 to 156, 23 to 59, and 0 to 42 days with the projected peaks being 1.7 to 9.4 (mean 4.1, SE 1.1), 1.2 to 7.0 (mean 3.5, SE 0.9), 1.2 to 1.8 (mean 1.4, SE 0.06), and 1.0 to 1.3 (mean 1.1, SE 0.04) times the observed peaks under the no-vaccination, partial vaccination, no-booster, and half-booster scenarios, respectively. Those indicate that the effect of the vaccination potentially avoided severe runs on the health system of the 7 countries.

Comparing the actual observations with the 3 scenarios regarding the booster shot taking, the peak values of active confirmed people in the 7 countries would increase by 23% to 78% (mean 43%, SE 6%) under the no-booster scenario and decrease by 26% to 63% (mean 41%, SE 5%) relative to the observed peaks under the twice booster scenario, which verifies that the booster doses can further relieve the pressure on the healthcare system in the Omicron era due to its higher VPRs against the Omicron variants as reported in Fig. 1.

It is noted that, in Italy, the projected peak under the partial vaccination scenario was higher than that under the no-vaccination scenario in the Intervening II period. This was due to the fact that a considerable proportion of population would have been infected in the Delta-dominated period under the no-vaccine scenario, and Italy had the highest rate of partial vaccination before the Intervening II period among the 7 countries (Fig. 3A). However, the immunity acquired from the partial vaccination gradually expired without further injected immunity from the full dose. This would lead to a rebound in the numbers of susceptibles, even exceeding those under the no-vaccination scenario as shown in Fig. S1. This result suggests the importance of acquiring additional immunity through full and booster doses.

## Discussion

This study targets the population protection rates of vaccines. Although the vaccine efficacies are different between different age groups, our results reveal the overall protection rates of a country, which are informative on the total infection size and the demand on the health resources of a country. It is shown that the real-world VPRs of the partial, full, and booster vaccination decreased with time. The full vaccination was effective before Omicron with the VPRs remaining above 50%, which became insufficient when the Omicron was dominant. The booster shot was effective in slowing down the epidemics in both the Delta- and Omicron-dominated periods with the average VPRs well above the 50% threshold. Our results on the real-world VPRs were consistent with the vaccine effectiveness in the existing cohort or case–control studies [9,12,21]. The necessity of the full and the booster vaccination is further highlighted by significant reductions in daily numbers of active confirmed cases in the scenario analyses.

Two sets of sensitivity analyses have been conducted to explore the impact of the uncertainty associated with model parameters for the daily asymptomatic rate 1 − *θ_t_* and the average time duration *μ_r_* from recovery to loss of natural immunity (average duration for reinfection) on the estimated VPRs. The sensitivity analyses show that the average absolute differences between the VPRs by using different values of the daily asymptomatic rate and the average duration for reinfection were 1.26% (SE: 0.23%) and 0.94% (SE: 0.17%), respectively, indicating the robustness of the estimated VPRs with respect to the 2 key parameters.

Despite the effectiveness of the booster shot in the Omicron era, the booster vaccine coverage had a rather slow pace of growth with less than 9% increase from 2022 March 15 to 2022 July 17 in Italy, Turkey, the UK, and the US, as shown in Fig. 3A. The booster coverage was 37.6% on 2022 July 17 in the US, which was only increased by 8.5% over the 4 months since 2022 March 15, and the UK’s increased by only 3.1% over the same period. Thus, there is ample room for vigorous promotion of booster shots in all countries to realize their benefits in reducing both the size of the epidemics and death. The encouraging effects of the booster shots also encourage consideration for another dose after the booster shot to cope with the continuing evolution of the SARS-CoV-2 viruses.

## Materials and Methods

We obtained the publicly available nationwide epidemiological and vaccination data from 2020 February 23 to 2022 March 15 for the 7 countries considered in this study. The daily cumulative numbers of the confirmed cases and deaths were obtained from “the 2019 Novel Coronavirus Visual Dashboard" at Johns Hopkins University. The daily recovered cases were imputed using 14 days as the average time of recovery from diagnosis by Eq. 3, since recoveries have not been reported since August for those countries. The information on the vaccine types and the cumulative numbers of people having received the partial, full, and booster vaccination was obtained from the official reports of the countries [22]. To reduce measurement errors, we used the kernel smoothing approach [20] to smooth the daily observed data before analysis.

The study period in this research is from the start of the vaccine in a country to 2022 March 15, while part of the pre-vaccine period was considered for model parameter estimation. For each country, we divide the post-vaccine era into 6 consecutive nonoverlapping periods: the pre-Delta period from the start of vaccination till the Delta variant was first detected in the country, the following intervening period (Intervening I) until the Delta variant became predominant (more than 50% of the daily detected cases), the Delta-dominated period when the majority of the cases were caused by the Delta variant till the start of booster shots, the pre-Omicron period from the start of booster shots till the Omicron variant was first detected, the intervening period (Intervening II) till Omicron became predominant, and the Omicron-dominated period when the majority of the cases were caused by the Omicron variant. It is noted that the dominant variant was still Delta in the pre-Omicron period in the 7 countries. Since the start of the booster shot (2021 June 21) was close to the date (2021 June 29) when the Delta variant began to dominate in Turkey, we merged its Delta-dominated period and pre-Omicron period, which resulted in Turkey having only 5 periods. The 6 key dates for determining the post-vaccine periods, including the start dates of vaccination and boosters, the dates for the first detection of the Delta and Omicron variants, and the dates when Delta and Omicron began to dominate, are provided in Table S2. The dominating variant at a time in a country is available in [23], while the dates for the first detection of the Delta and Omicron variants for the 7 countries were collected from [24,25]. The Delta and Omicron dominant dates were decided as the first dates when the proportions of Delta and Omicron exceeded 50% in all SARS-CoV-2 viruses by genome sequencing, respectively.

We estimate the real-world VPR by building a stochastic epidemiological model with 11 compartments to quantify the epidemic process and developing a novel estimation procedure for its parameters.

### Stochastic epidemiological model

**Fig. 4.**
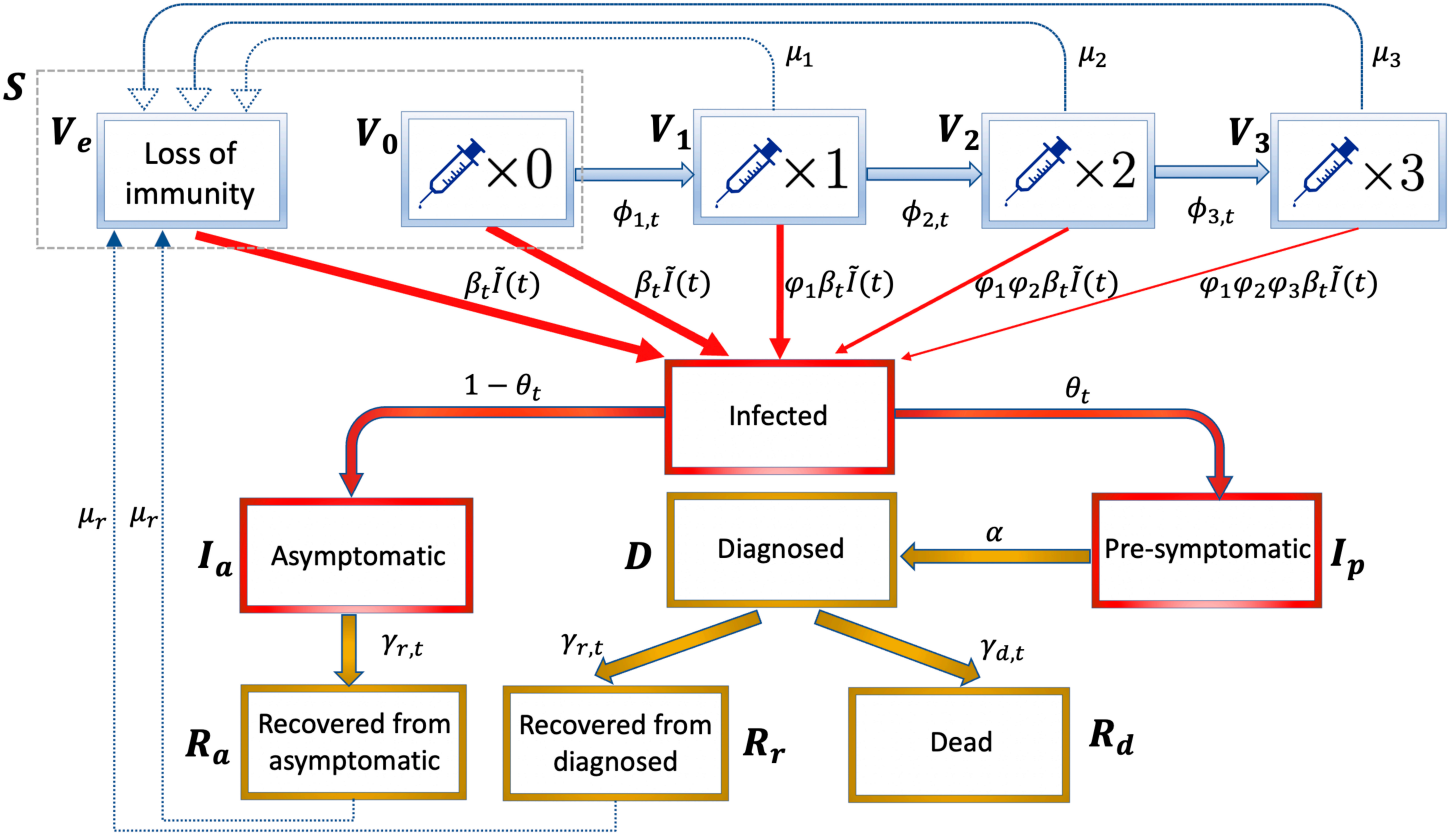
Compartments and their structure flows of the proposed epidemiological model, where the unvaccinated and uninfected people *V*_0_ and the vaccinated and uninfected people with expired vaccine immunity and the recovered with expired natural immunity *V_e_* constitute state *S* = *V*_0_ + *V_e_* of currently uninfected people without immunity, and *φ*_1_, *φ*_2_, and *φ*_3_ are the parameters representing vaccine protection rates.

The stochastic epidemiological model describes the spread of the SARS-CoV-2 virus and the daily increase of infected cases in a country. The compartments and flows between compartments are shown in Fig. 4. The model allows nonpermanent vaccine and natural immunity, breakthroughs in vaccinated people, and being asymptomatic and infectious before clinical diagnosis (pre-symptomatic). The susceptible population is divided into 5 uninfected compartments, the ones with no (*V*_0_), partial (*V*_1_), full (*V*_2_), and booster (*V*_3_) vaccine immunity and the ones who have been vaccinated but lost vaccine immunity or have recovered from previous infection but lost natural immunity (*V_e_*), in the top row of Fig. 4. The currently uninfected compartment without vaccine or natural immunity (*S*) consists of unvaccinated people (*V*_0_), the vaccinated people with expired vaccine immunity, and the recovered with expired natural immunity (*V_e_*). Let *ϕ*_1,*t*_, *ϕ*_2,*t*_, and *ϕ*_3,*t*_ be the time-varying vaccination rates from *V*_0_(*t*) to *V*_1_(*t*), *V*_1_(*t*) to *V*_2_(*t*), and *V*_2_(*t*) to *V*_3_(*t*), respectively, at day *t*. We consider the temporary vaccine-induced immunity and natural immunity with the average lengths of immunity after the partial, full, and booster doses of vaccines and the recovery from previous infection being 1/*μ*_1_, 1/*μ*_2_, 1/*μ*_3_, and 1/*μ_r_* days, respectively. We set *μ*_1_ = 1/60, *μ*_2_ = 1/240, *μ*_3_ = 1/300 and *μ*_1_ = 1/56, *μ*_2_ = 1/90, *μ*_3_ = 1/140 before and after the emergence of the Omicron variant according to the existing studies [26–31]. Motivated by the study that shows that SARS-CoV-2 reinfections were uncommon (less than 1% of the total confirmed infections) until the end of 2021 [32], we set *μ_r_* = 0 before the emergence of the Omicron variant, and set *μ_r_* = 1/480 after the emergence of the Omicron variant in the main analysis and *μ_r_* = 1/180 in the sensitivity analysis based on studies [33–35] on the duration of immune protection from infection.

The uninfected individuals may catch the virus by making contact with the infected ones, which are divided into 3 compartments: asymptomatic (*I_a_*), pre-symptomatic (*I_p_*), and diagnosed (*D*), with the time-varying infection rates $\beta_{t}^{I_{a}}$, *β_t_*, and $\beta_{t}^{D}$, respectively. Asymptomatic cases represent the ones that show no symptoms and do not take a test. The pre-symptomatic period stands for the period after infection but before laboratory confirmation. The pre-symptomatic cases would be diagnosed at the rate *α* ∈ (0, 1), where 1/*α* represents the average days between being infected and laboratory diagnosis.

Following the setup of the infection rates for different stages of infections [36], we assume that the pre-symptomatic compartment is 5 times more infectious than the asymptomatic and diagnosed compartments, namely, $\beta_{t}^{I_{a}}=\beta_{t}^{D}=\beta_{t}/\zeta$ with *ζ* = 5, as diagnosed cases would take precautions and quarantine at home, and asymptomatic cases have no symptoms and should be less infectious. Let *M* be the total population size, and $\tilde{I}\left(t\right)$ be the standardized total infection load with respect to the time-varying infection rate of the pre-symptomatic cases *β_t_*, which is equal to the size of the pre-symptomatic compartment divided by *M* plus those of the asymptomatic and diagnosed compartments divided by 5*M*, namely

$$\tilde{I}\left(t\right)=\left\{I_{a}\left(t\right)/5+I_{p}\left(t\right)+D\left(t\right)/5\right\}/M.$$

Allowing time-varying infection rate (*β_t_*) is needed as government and citizens’ responses to COVID-19 change over time and the virus itself keeps mutating. We assume that the daily new infections from the susceptible groups without vaccine or natural immunity, and with partial, full, and booster vaccine immunity follow conditional Poisson distributions with means equal to $\beta_{t}\tilde{I}\left(t\right)$, $\varphi_{1}\beta_{t}\tilde{I}\left(t\right)$, $\varphi_{1}\varphi_{2}\beta_{t}\tilde{I}\left(t\right)$, and $\varphi_{1}\varphi_{2}\varphi_{3}\beta_{t}\tilde{I}\left(t\right)$ multiplying the size of the corresponding group, respectively, which are shown in Fig. 4. The vaccine effects are reflected by *φ*_1_, *φ*_2_, *φ*_3_ ∈ (0, 1), where the individuals having partial, full, and booster vaccine immunity are less likely to be infected compared to those without vaccine immunity by the factors *φ*_1_, *φ*_1_*φ*_2_ and *φ*_1_*φ*_2_*φ*_3_, respectively. The VPRs for the 3 vaccine compartments are 1 − *φ*_1_, 1 − *φ*_1_*φ*_2_, and 1 − *φ*_1_*φ*_2_*φ*_3_, respectively.

All infected people will develop either asymptomatic *I_a_* or symptomatic *I_p_*. A new infection has 1 − *θ_t_* probability of being asymptomatic, which is modeled by a binomial distribution. As existing studies found 20% of COVID-19 infections were asymptomatic at the early stage of the COVID-19 pandemic [37], 40% before the detection of Omicron [38], and 80% to 90% for Omicron [39], we assume *θ_t_* to be a piecewise linear function, which is 0.8 till the first detection of the Delta variant, then linearly decreases to 0.6 till the first detection of the Omicron variant, and then till 2022 March 15 linearly decreases to 0.1 in the main analysis and to 0.2 in the sensitivity analysis. All asymptomatic infections are never diagnosed and will recover naturally *R_a_* with the recovery rate *γ*_*r*,*t*_. The pre-symptomatic cases will be confirmed and moved to the diagnosed state *D* with the diagnosis rate *α* in a future date, then to the recovered *R_r_* with the recovery rate *γ*_*r*,*t*_ and the dead *R_d_* with the death rate *γ*_*d*,*t*_. Let Δ denote the daily change of a compartment. Recall that *S*(*t*) = *V*_0_(*t*) + *V_e_*(*t*). The relationship of the conditional means of the aforementioned compartments at time *t* is presented in Eq. 1, where $F_{t}$ denotes the sigma-algebra for all the information up to time *t*.

$$\begin{array}{c} E\left\{\Delta V_{0}\left(t\right)|F_{t}\right\}=-\beta_{t}\tilde{I}\left(t\right)V_{0}\left(t\right)-\phi_{1,t}V_{0}\left(t\right)\\ E\left\{\Delta V_{e}\left(t\right)|F_{t}\right\}=-\beta_{t}\tilde{I}\left(t\right)V_{e}\left(t\right)+\mu_{1}V_{1}\left(t\right)+\mu_{2}V_{2}\left(t\right)+\mu_{3}V_{3}\left(t\right)+\mu_{r}R_{r}\left(t\right)+\mu_{r}R_{a}\left(t\right),\\ E\left\{\Delta V_{1}\left(t\right)|F_{t}\right\}=\phi_{1,t}V_{0}\left(t\right)-\mu_{1}V_{1}\left(t\right)-\varphi_{1}\beta_{t}\tilde{I}\left(t\right)V_{1}\left(t\right)-\phi_{2,t}V_{1}\left(t\right),\\ E\left\{\Delta V_{2}\left(t\right)|F_{t}\right\}=\phi_{2,t}V_{1}\left(t\right)-\mu_{2}V_{2}\left(t\right)-\varphi_{1}\varphi_{2}\beta_{t}\tilde{I}\left(t\right)V_{2}\left(t\right)-\phi_{3,t}V_{2}\left(t\right),\\ E\left\{\Delta V_{3}\left(t\right)|F_{t}\right\}=\phi_{3,t}V_{2}\left(t\right)-\mu_{3}V_{3}\left(t\right)-\varphi_{1}\varphi_{2}\varphi_{3}\beta_{t}\tilde{I}\left(t\right)V_{3}\left(t\right),\\ E\left\{\Delta I_{a}\left(t\right)|F_{t}\right\}=\left(1-\theta_{t}\right)\beta_{t}\tilde{I}\left(t\right)\left\{S\left(t\right)+\varphi_{1}V_{1}\left(t\right)+\varphi_{1}\varphi_{2}V_{2}\left(t\right)+\varphi_{1}\varphi_{2}\varphi_{3}V_{3}\left(t\right)\right\}-\gamma_{r,t}I_{a}\left(t\right),\\ E\left\{\Delta I_{p}\left(t\right)|F_{t}\right\}=\theta_{t}\beta_{t}\tilde{I}\left(t\right)\left\{S\left(t\right)+\varphi_{1}V_{1}\left(t\right)+\varphi_{1}\varphi_{2}V_{2}\left(t\right)+\varphi_{1}\varphi_{2}\varphi_{3}V_{3}\left(t\right)\right\}-\alpha I_{p}\left(t\right),\\ E\left\{\Delta D\left(t\right)|F_{t}\right\}=\alpha I_{p}\left(t\right)-\left(\gamma_{r,t}+\gamma_{d,t}\right)D\left(t\right),E\left\{\Delta R_{a}\left(t\right)|F_{t}\right\}=\gamma_{r,t}I_{a}\left(t\right)-\mu_{r}R_{a}\left(t\right),\\ E\left\{\Delta R_{r}\left(t\right)|F_{t}\right\}=\gamma_{r,t}D\left(t\right)-\mu_{r}R_{r}\left(t\right)\;\text{and}\;E\left\{\Delta R_{d}\left(t\right)|F_{t}\right\}=\gamma_{d,t}D\left(t\right). \end{array}$$ (1)

The proposed stochastic epidemic model assumes that the daily increments of those compartments follow Poisson distributions with the conditional means specified by the equations in Eq. 1. It can be shown that the effective reproduction number *R_t_* under the proposed model is Eq. 2,

$$\begin{array}{c} R_{t}=\left\{\left(1-\theta_{t}\right)\frac{\beta_{t}^{I_{a}}}{\gamma_{r,t}}+\theta_{t}\left(\frac{\beta_{t}}{\alpha }+\frac{\beta_{t}^{D}}{\gamma_{r,t}+\gamma_{d,t}}\right)\right\}\\ \frac{S\left(t\right)+\varphi_{1}V_{1}\left(t\right)+\varphi_{1}\varphi_{2}V_{2}\left(t\right)+\varphi_{1}\varphi_{2}\varphi_{3}V_{3}\left(t\right)}{M}. \end{array}$$ (2)

The effective reproduction number is a key epidemiological parameter. When *R_t_* > 1(<1), the epidemic is increasing (decreasing).

### Estimation

Note that the observed data of a country are ${\left\{N\left(t\right),R_{d}\left(t\right),G_{1}\left(t\right),G_{2}\left(t\right),G_{3}\left(t\right)\right\}}_{t=1}^{T}$, where *N*(*t*), *R_d_*(*t*), *G*_1_(*t*), *G*_2_(*t*), and *G*_3_(*t*) are the daily cumulative numbers of the confirmed cases, deaths, and the partial, full and booster vaccinated people. Since the recovery data were only reported at the beginning of the pandemic, and the average recovery time since diagnosis was 14 days suggested by Guan et al. [40], we first impute the numbers of recovery (when it was not available) with the recovery rate *γ*_*r*,*t*_ set as 1/14 and impute the active confirmed infections by

R^rt=R^rt−1+D^t−1/14−μrR^rt−1,D^t=D^t−1+ΔNt−1−D^t−1/14−ΔRdt−1. (3)

Following a multi-step multi-time range procedure developed in Zhu et al. [41], we estimate the diagnosis rate *α*, and the VPR parameters *φ*_1_, *φ*_2_, and *φ*_3_ of a country via minimizing certain criterion functions using different periods of data for different variants of SARS-CoV-2 virus, and time-varying infection *β_t_*, recovery *γ*_*r*,*t*_, and death rates *γ*_*d*,*t*_ in the proposed model via a nonparametric regression method, which leads to the estimation of the effective reproduction number *R_t_* via Eq. 2 and the VPRs 1 − *φ*_1_, 1 − *φ*_1_*φ*_2_, and 1 − *φ*_1_*φ*_2_*φ*_3_ for the partial, full, and booster shots of a country. Simulation experiments for evaluating the accuracy of the estimation approach under similar stochastic epidemiological models had been made in 2 studies [20,41], which showed good performance of the estimation method.

#### Estimation of removal rates *γ*_*d*,*t*_ and *γ*_*r*,*t*_

From the last 2 equations in Eq. 1, we estimate *γ*_*d*,*t*_ and *γ*_*r*,*t*_ by local linear regression of the daily new deaths Δ*R_d_*(*t*) and daily new recoveries Δ*R_r_*(*t*) on daily active confirmed infections *D*(*t*) via

$$\begin{array}{cc} {\hat{\gamma }}_{d,t} & =\frac{\sum_{i=1}^{T-1}‍D\left(i\right)\Delta R_{d}\left(i\right)B\left(\left(t-i\right)/h_{d}\right)}{\sum_{i=1}^{T-1}‍D{\left(i\right)}^{2}B\left(\left(t-i\right)/h_{d}\right)}\;\text{and}\\ {\hat{\gamma }}_{r,t} & =\frac{\sum_{i=1}^{T-1}‍D\left(i\right)\Delta R_{r}\left(i\right)B\left(\left(t-i\right)/h_{r}\right)}{\sum_{i=1}^{T-1}‍D{\left(i\right)}^{2}B\left(\left(t-i\right)/h_{r}\right)}, \end{array}$$ (4)

where *B*(·) is a kernel function, and *h_d_* and *h_r_* are the temporal smoothing bandwidths [42].

#### Estimation of diagnosis rate *α*

We use the time period $S_{1}$ of 1 month right before the start of public vaccination to estimate the diagnosis rate *α*. In this period, there were no vaccine effects, where *φ*_1_ = *φ*_2_ = *φ*_3_ = 1 and *G*_1_(*t*) = *G*_2_(*t*) = *G*_3_(*t*) = 0. Given *α* and *β_t_*, we consider a contrast measure between the estimated size of the pre-symptomatic compartment *I_p_*(*t*) based on the observed data and that simulated from the proposed model, in the form of

f1αβt=1S1∑t∈S1E^αIptFt−1/I^pαt−12forI^pαt=ΔNt/α, (5)

where Δ*N*(*t*)/*α* stands for an imputation for *I_p_*(*t*) based on the new confirmed cases Δ*N*(*t*) on day *t* at a given *α*, and ${\hat{E}}^{\alpha }\left\{I_{p}\left(t\right)|F_{t-1}\right\}$ is a simulation-based estimate of the conditional expectation of *I_p_*(*t*) given data up to *t* − 1 by averaging the simulated trajectories under the proposed model using the given *α* and *β_t_*. The nonlinear infection rate *β_t_* is approximated by B-spline functions. We minimize this contrast measure *f*_1_(*α*, *β_t_*) with respect to *α* and the coefficients of B-spline basis functions of *β_t_* by the grid search algorithm. This estimation approach can be viewed as a minimum distance method that minimizes the distance between the trajectories implied by the model and the observed data of daily new confirmed cases. However, due to the unobservable compartments, imputation is needed.

#### Estimation of vaccine effects *φ*_1_, *φ*_2_, and *φ*_3_

For each of the 6 periods with different COVID-19 variants (listed in Table S3), we estimate the VPR parameters *φ*_1_, *φ*_2_, and *φ*_3_ by a similar method as the estimation of *α*. Note that we set *φ*_3_ = 1 for the periods before the start of booster shot vaccination. Similar to the objective function *f*_1_(*α*, *β_t_*) in Eq. 5, we consider to minimize

$$\begin{array}{c} f_{2}(\varphi_{1},\varphi_{2},\varphi_{3},\beta_{t})=\\ \frac{1}{|S_{2}|}\sum_{t\in S_{2}}‍\{{\hat{E}}^{\hat{\alpha },\varphi_{1},\varphi_{2},\varphi_{3}}\{I_{p}(t)|F_{t-1}\}/{\hat{I}}_{p}^{\hat{\alpha }}(t)-1{\}}^{2}, \end{array}$$ (6)

over a time range $S_{2}$, where ${\hat{I}}_{p}^{\hat{\alpha }}\left(t\right)=\Delta N\left(t\right)/\hat{\alpha }$ is the imputed value of *I_p_*(*t*) by the estimated diagnosis rate $\hat{\alpha }$ obtained in the previous step, and ${\hat{E}}^{\hat{\alpha },\varphi_{1},\varphi_{2},\varphi_{3}}\left\{I_{p}\left(t\right)|F_{t-1}\right\}$ is the estimated expectation of *I_p_*(*t*) by the simulations from the proposed model using the given parameters. The estimates of *φ*_1_, *φ*_2_, and *φ*_3_ are obtained by minimizing *f*_2_(*φ*_1_, *φ*_2_, *φ*_3_, *β_t_*) via the grid search algorithm and B-spline approximation of *β_t_*.

#### Estimation of infection rate *β_t_*

As the B-spline estimate of *β_t_* via optimizing the objective functions may not be continuous between the 6 study periods, we use kernel smoothing method for estimating *β_t_* after obtaining the estimates of *α*, *φ*_1_, *φ*_2_, and *φ*_3_, which is in a similar manner as the smoothing estimates ${\hat{\gamma }}_{d,t}$ and ${\hat{\gamma }}_{r,t}$ in Eq. 4.

Note that ${\hat{I}}_{p}^{\hat{\alpha }}\left(t\right)=\Delta N\left(t\right)/\hat{\alpha }$ is the imputed value of *I_p_*(*t*). We can impute *I_a_*(*t*) and *R_a_*(*t*) as

$$\begin{array}{c} {\hat{I}}_{a}^{\alpha }\left(t\right)=\left\{\Delta {\hat{I}}_{p}^{\alpha }\left(t-1\right)+\alpha {\hat{I}}_{p}^{\alpha }\left(t-1\right)\right\}\left(1-\theta_{t-1}\right)/\theta_{t-1}\\ +\left(1-{\hat{\gamma }}_{r,t-1}\right){\hat{I}}_{a}^{\alpha }\left(t-1\right)\;\text{and}\\ {\hat{R}}_{a}^{\alpha }\left(t\right)={\hat{R}}_{a}^{\alpha }\left(t-1\right)+{\hat{\gamma }}_{r,t-1}{\hat{I}}_{a}^{\alpha }\left(t-1\right)-\mu_{r}{\hat{R}}_{a}^{\hat{\alpha }}\left(t-1\right), \end{array}$$

respectively. As {Δ*I_p_*(*t*) + *αI_p_*(*t*)}/[*θ_t_*{*S*(*t*) + *φ*_1_*V*_1_(*t*) + *φ*_1_*φ*_2_*V*_2_(*t*) + *φ*_1_*φ*_2_*φ*_3_*V*_3_(*t*)}] serves as a substitution for $\beta_{t}\tilde{I}\left(t\right)$, we can impute *V*_1_(*t*), *V*_2_(*t*), *V*_3_(*t*), and *S*(*t*) by

$$\begin{array}{c} {\hat{V}}_{1}^{\hat{\alpha }}\left(t\right)=\left(1-\mu_{1}\right){\hat{V}}_{1}^{\hat{\alpha }}\left(t-1\right)-\hat{r}\left(t-1\right){\hat{\varphi }}_{1}{\hat{V}}_{1}^{\hat{\alpha }}\left(t-1\right)\\ +\Delta G_{1}\left(t-1\right)-\Delta G_{2}\left(t-1\right),\\ {\hat{V}}_{2}^{\hat{\alpha }}\left(t\right)=\left(1-\mu_{2}\right){\hat{V}}_{2}^{\hat{\alpha }}\left(t-1\right)-\hat{r}\left(t-1\right){\hat{\varphi }}_{1}{\hat{\varphi }}_{2}{\hat{V}}_{2}^{\hat{\alpha }}\left(t-1\right)\\ +\Delta G_{2}\left(t-1\right)-\Delta G_{3}\left(t-1\right),\\ {\hat{V}}_{3}^{\hat{\alpha }}\left(t\right)=\left(1-\mu_{3}\right){\hat{V}}_{3}^{\hat{\alpha }}\left(t-1\right)-\hat{r}\left(t-1\right){\hat{\varphi }}_{1}{\hat{\varphi }}_{2}{\hat{\varphi }}_{3}{\hat{V}}_{3}^{\hat{\alpha }}\left(t-1\right)+\Delta G_{3}\left(t-1\right),\\ {\hat{S}}^{\hat{\alpha }}\left(t\right)={\hat{S}}^{\hat{\alpha }}\left(t-1\right)-\hat{r}\left(t-1\right){\hat{S}}^{\hat{\alpha }}\left(t-1\right)-\Delta G_{1}\left(t-1\right)+\mu_{1}{\hat{V}}_{1}^{\hat{\alpha }}\left(t-1\right)\\ +\mu_{2}{\hat{V}}_{2}^{\hat{\alpha }}\left(t-1\right)+\mu_{3}{\hat{V}}_{3}^{\hat{\alpha }}\left(t-1\right)+\mu_{r}{\hat{R}}_{r}\left(t-1\right)+\mu_{r}{\hat{R}}_{a}^{\hat{\alpha }}\left(t-1\right). \end{array}$$

with the initial values of *V*_1_, *V*_2_, and *V*_3_ being zero at the start of the vaccination, where

$$\hat{r}\left(t\right)=\frac{{\hat{I}}_{p}^{\hat{\alpha }}\left(t+1\right)-\left(1-\hat{\alpha }\right){\hat{I}}_{p}^{\hat{\alpha }}\left(t\right)}{\theta_{t}\left({\hat{S}}^{\hat{\alpha }}\left(t\right)+{\hat{\varphi }}_{1}{\hat{V}}_{1}^{\hat{\alpha }}\left(t\right)+{\hat{\varphi }}_{1}{\hat{\varphi }}_{2}{\hat{V}}_{2}^{\hat{\alpha }}\left(t\right)+{\hat{\varphi }}_{1}{\hat{\varphi }}_{2}{\hat{\varphi }}_{3}{\hat{V}}_{3}^{\hat{\alpha }}\left(t\right)\right)}.$$

The infection rate *β_t_* can be estimated by nonparametric regression of $Y\left(t\right)=\left\{{\hat{I}}_{p}^{\hat{\alpha }}\left(t+1\right)+\left(\hat{\alpha }-1\right){\hat{I}}_{p}^{\hat{\alpha }}\left(t\right)\right\}/\theta_{t}$ on $X\left(t\right)=\left[{\hat{I}}_{p}^{\hat{\alpha }}\left(t\right)+\left\{D\left(t\right)+{\hat{I}}_{a}^{\hat{\alpha }}\left(t\right)\right\}/\zeta \right]\left\{{\hat{S}}^{\hat{\alpha }}\left(t\right)+{\hat{\varphi }}_{1}{\hat{V}}_{1}^{\hat{\alpha }}\left(t\right)+{\hat{\varphi }}_{1}{\hat{\varphi }}_{2}{\hat{V}}_{2}^{\hat{\alpha }}\left(t\right)+{\hat{\varphi }}_{1}{\hat{\varphi }}_{2}{\hat{\varphi }}_{3}{\hat{V}}_{3}^{\hat{\alpha }}\left(t\right)\right\}/M$ as

$${\hat{\beta }}_{t}=\frac{\sum_{i=1}^{T-2}‍X\left(i\right)Y\left(i\right)B\left\{\left(t-i\right)/h\right\}}{\sum_{i=1}^{T-2}‍X{\left(i\right)}^{2}B\left\{\left(t-i\right)/h\right\}}.$$ (7)

For pre-vaccine eras, *β_t_* is the same estimated as Eq. 7 with ${\hat{V}}_{1}^{\hat{\alpha }}\left(t\right)$, ${\hat{V}}_{2}^{\hat{\alpha }}\left(t\right)$, and ${\hat{V}}_{3}^{\hat{\alpha }}\left(t\right)$ set as zero.

### Parametric bootstrap inference

Bootstrap procedure is used to obtain confidence intervals for the estimated parameters concerned in the aforementioned 4 steps of the estimation. Given the estimates ${\hat{\varphi }}_{1}$, ${\hat{\varphi }}_{2}$, and ${\hat{\varphi }}_{3}$ from each of the 6 post-vaccine periods, and $\hat{\alpha }$, ${\hat{\beta }}_{t}$, ${\hat{\gamma }}_{r,t}$, and ${\hat{\gamma }}_{d,t}$, we generate bootstrap resampled trajectories based on proposed stochastic epidemic model. All the parameters were re-estimated based on the bootstrap resampled observations. The resampling was replicated for a large number (*B*) of times to obtain *B* independent bootstrap estimates for the parameter. The sample standard deviation and the 2.5% and 97.5% percentiles of the bootstrap estimates can be used to estimate the standard error of the estimates obtained in the above 4 steps of the estimation and to construct confidence intervals for the parameters.

### Fitting performance

We use the relative errors between the mean projected numbers of cumulative confirmed cases by 1,000 simulations with the estimated parameters and the observed numbers of cumulative confirmed cases to evaluate the fitting performance of our proposed model. The relative errors in the main analysis of the 7 countries shown in Fig. S3 were not higher than 25%, which reflects that our model performs well.

### Sensitivity analysis

The number of reported cases is influenced by the severity of symptoms, public willingness to do testing, and the testing capacity. These factors determine the pre-symptomatic proportion *θ_t_* and the diagnosis rate *α* of the pre-symptomatic cases in our model. While *α* is empirically estimated by the proposed method, *θ_t_* is determined from existing studies. Murray [39] suggested that the asymptomatic cases accounted for 80% to 90% of COVID-19 infections in the Omicron era. In the main analysis, the probability for new infections being asymptomatic since the detection of Omicron variant was assumed to increase linearly from 40% to 90%. To explore the sensitivity of this specification, we conducted a sensitivity analysis that assumed the probability of being asymptomatic for new infections after the Omicron increased linearly from 40% to 80%. The estimated VPRs in the Intervening II and the Omicron-dominated periods for the 7 countries are reported in Table S7, which shows that the magnitude of the differences between VPRs by altering the daily asymptomatic rates 1 − *θ_t_* was no more than 8.6% and the average of the absolute differences was 1.26% (SE: 0.23%). We have also conducted a sensitivity analysis on the estimated VPRs with respect to *μ_r_*, the time duration from recovery to loss of natural immunity. It shows that the differences between the estimated VPRs in the 7 countries with *μ_r_* being 16 months as in the main analysis and those with *μ_r_* being 6 months were at most 5% apart with the average absolute differences being 0.94% (SE: 0.17%). Table S7 contains the details for the 2 sets of sensitivity analyses.

### Scenario analysis

SA is conducted to evaluate the impacts of different vaccination strategies on the confirmed cases and death of the pandemic. Five vaccination scenarios were designed: (a) no vaccination at all; (b) receiving the partial but no full vaccination; (c) receiving the partial and full vaccination but no booster shots; receiving the booster shots only at half (d) and twice (e) of the actual daily booster vaccination rate.

Specifically, in the no- and partial vaccination scenarios (a and b) designed for the effects of the full vaccination, simulations were performed from the start of vaccination to 2022 March 15. During this period, the sub-population having received the full and booster doses of vaccines were set to zero while maintaining the actual numbers for having received partial vaccination in the partial vaccination scenario, and the sub-population receiving at least one dose was set to zero in the no-vaccination scenario. Similarly, for the last 3 scenarios (c to e) designed for evaluating the effects of the booster shot, simulations were performed from the start of the booster vaccination with the daily observed numbers of people having received the partial and full vaccination, while the numbers of people having received the booster doses were set to be zero, half and twice the observed vaccine up-takes, respectively. In the twice booster up-take scenario, the would-be numbers of people having received booster doses were truncated to the numbers of full vaccination at the time if the doubling up exceeds the latter number of the country. Under each of the scenarios, the daily adjusted numbers of vaccinated people and the empirically estimated VPRs of partial, full, and booster vaccination $1-{\hat{\varphi }}_{1}$, $1-{\hat{\varphi }}_{1}{\hat{\varphi }}_{2}$, and $1-{\hat{\varphi }}_{1}{\hat{\varphi }}_{2}{\hat{\varphi }}_{3}$ shown in Fig. 1 and Table S3, diagnosis rates $\hat{\alpha }$ in Table S2, and recovery rates ${\hat{\gamma }}_{r,t}$, death rates ${\hat{\gamma }}_{d,t}$, and infection rates ${\hat{\beta }}_{t}$ in Fig. S4 are plugged into the proposed epidemic model (Eq. 1) to project the would-be dynamics of the pandemic.

## Data Availability

The daily epidemiological and vaccination data that support the findings of this study are available in a public repository “Our World in Data” at https://covid.ourworldindata.org/. The codes used in this study are available on GitHub at https://github.com/zyrstat/Estimating-VPRs.

## Supplementary Materials

Supplementary Materials Fig. S1. Fig. S2. Fig. S3. Fig. S4. Table S1. Table S2. Table S3. Table S4. Table S5. Table S6. Table S7.
