# Supplementary material for: Real-World COVID-19 Vaccine Protection Rates against Infection in the Delta and Omicron Eras
Source: Research (Wash D C). 2023 Apr 5;6:0099. doi: 10.34133/research.0099 (PMC10202182; doi:10.34133/research.0099)
Supplement: Supplementary Materials — Fig. S1. Fig. S2. Fig. S3. Fig. S4. Table S1. Table S2. Table S3. Table S4. Table S5. Table S6. Table S7. [file research.0099.f1.pdf]

Supporting Information for "Real-World COVID-19 Vaccine Protection Rates against Infection in the Delta and Omicron Eras"

Yuru Zhu[1], Jia Gu[1], Yumou Qiu[2,*], and Song Xi Chen[1,3,*]

[1]Center for Statistical Science, Peking University.
[2]Department of Statistics, Iowa State University.
[3]School of Mathematical Science and Guanghua School of Management, Peking University.
[*]Corresponding author. Email: yumouqiu@iastate.edu, csx@gsm.pku.edu.cn

December 25, 2022

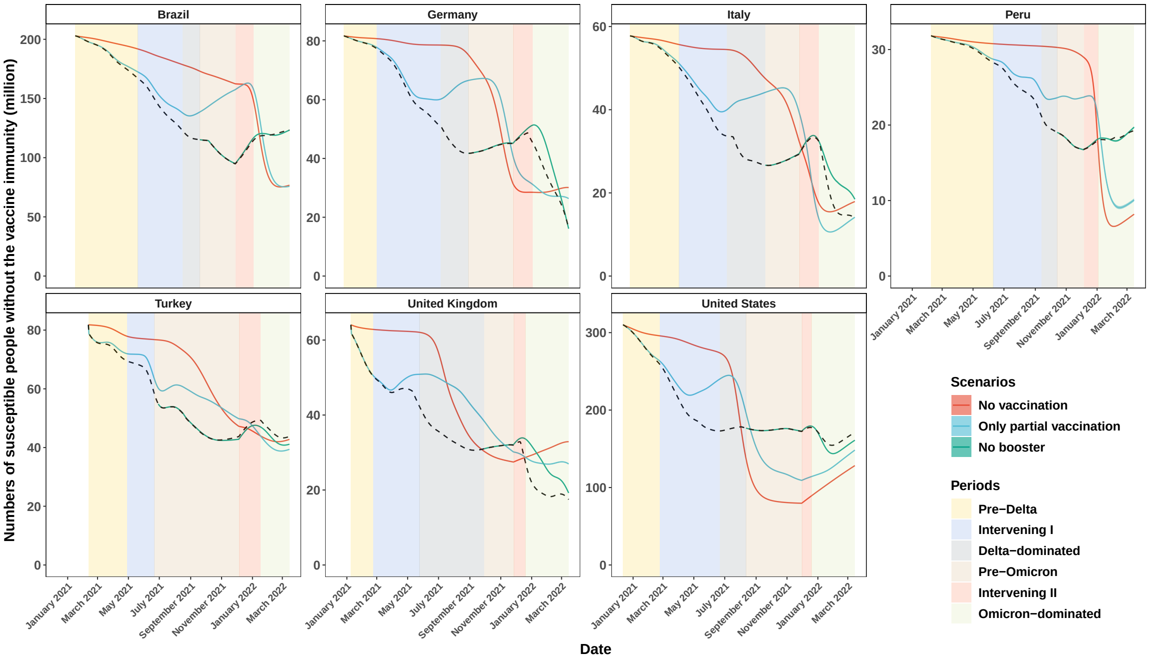

Figure S1: The projected daily numbers (in millions) of susceptible people with no vaccine immunity under the three vaccination scenarios (color curves) and the imputed ones using real data (black dashed lines). The 95% confidence bands of the projected numbers are indicated by colored areas.

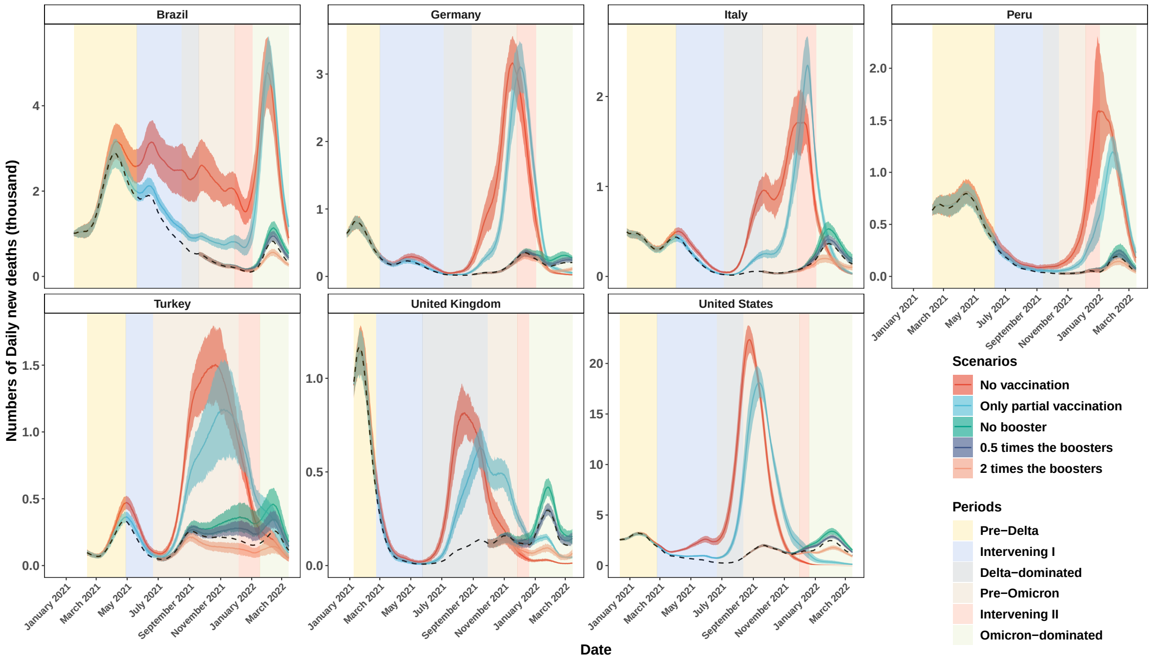

Figure S2: The actual (black dashed lines), and the projected numbers (in thousands) of daily new deaths (color curves) and their 95% confidence bands (color area) under the five vaccination scenarios.

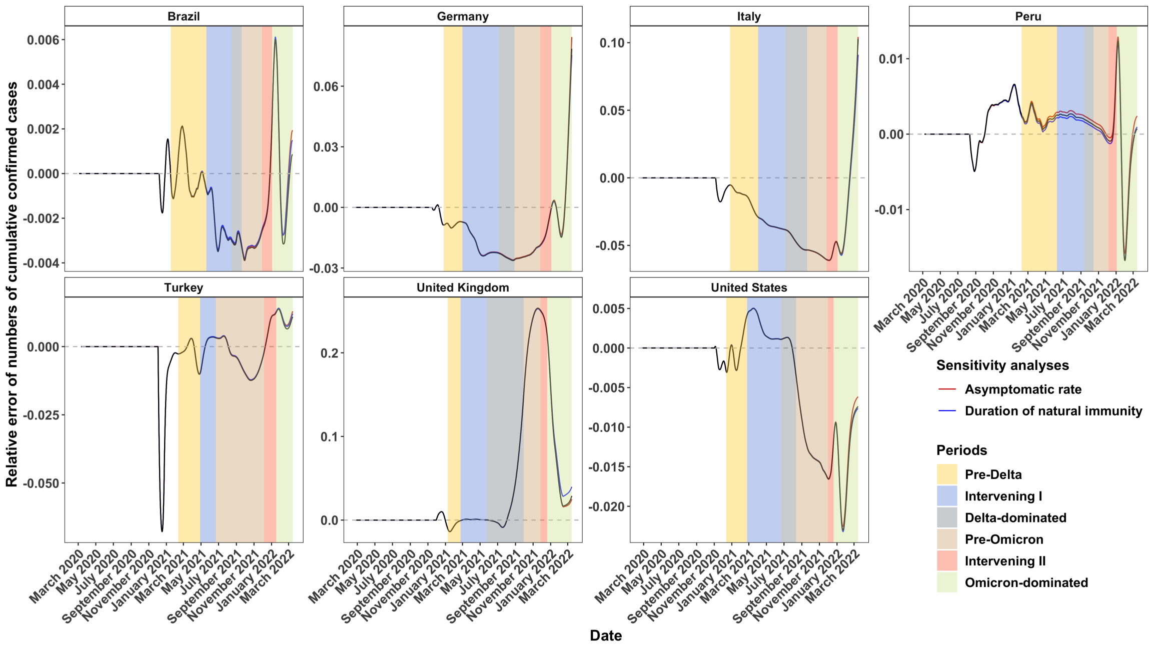

Figure S3: The relative errors between the mean projected numbers of cumulative confirmed cases by 1000 simulations with the estimated parameters and the observed numbers of cumulative confirmed cases for the main analysis (black), and sensitivity analyses for the asymptomatic rate (red) and the duration of natural immunity (blue) in the seven countries.

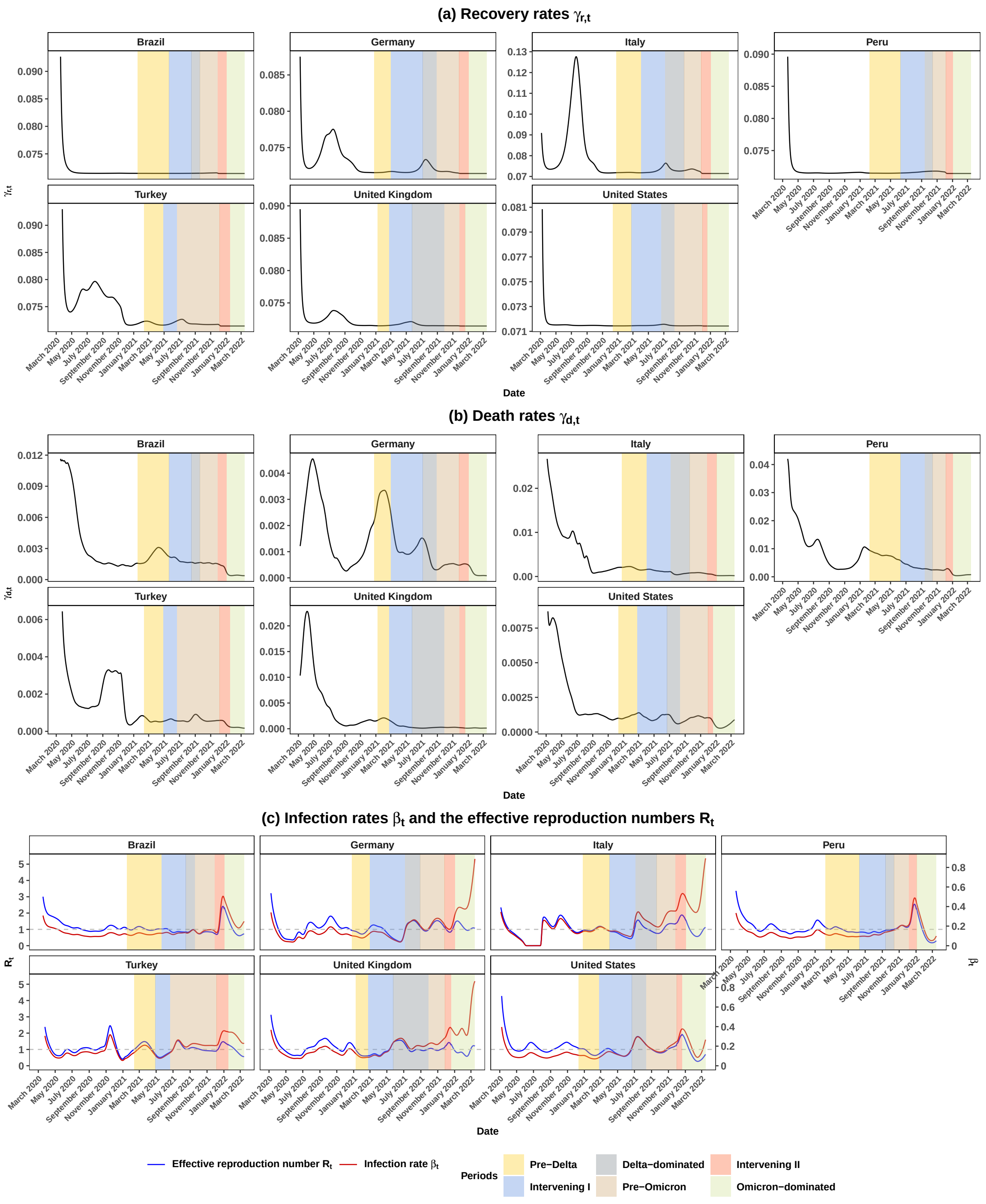

Figure S4: Curves of the estimated (a) recovery rates $\gamma_{r,t}$, (b) death rates $\gamma_{d,t}$, (c) infection rates β_t (red) and the effective reproduction numbers R_t (blue).

Table S1: The vaccine efficacy (for clinical trials) or vaccine effectiveness (for observational studies) and corresponding 95% confidence interval (in parentheses) obtained in recent studies. Observational studies were more common in the Omicron era. A "–" indicates the primary vaccination of Janssen is one dose.

(a) Vaccine efficacy or effectiveness of one or two doses.

Vaccine name (type)	Original strain		Delta variant		Omicron variant	
	one dose	two doses	one dose	two doses	one dose	two doses
Pfizer (mRNA)	52 (29.5 - 68.4)[1]	95 (90.3 - 97.6)[1]	45.2 (43.3 - 47.1)[2]	90.9 (89.6 - 92.0)[2]	42.8 (40.3 - 45.1)[2]	65.5 (63.9 - 67.0)[2]
Moderna (mRNA)	95.2 (91.2 - 97.4)[3]	94.1 (89.3 - 96.8)[3]	60.1 (51.8 - 66.9)[2]	94.5 (90.5 - 96.9)[2]	47.9 (43.1 - 52.3)[2]	75.1 (70.8 - 78.7)[2]
Janssen (viral vector)	66.9 (59.0 - 73.4)[4]	–	60[5]	–	24 (18 - 29)[6]	–
AstraZeneca (viral vector)	64.1 (50.5 - 73.9)[7]	62.1 (41.0 - 75.7)[7]	42.9 (39.8 - 45.9)[2]	82.8 (74.5 - 88.4)[2]	17.7 (14.3-21.0)[2]	48.9 (39.2 - 57.1)[2]
	76.0 (59.3 - 85.9)[8]	66.7 (57.4 - 74.0)[8]	30.0 (24.3 - 35.3)[9]	67.0 (61.3 - 71.8)[9]		
Sinopharm (inactivated virus)	65.5 (52.0 - 75.1)[10]	78.1 (64.8 - 86.3)[10]	13.8 (-60.2 - 54.8)[11]	59.0 (16.0 - 81.6)[11]		
Sinovac (inactivated virus)	57.9 (46.4 - 66.9) [12]	50.7 (36.0 - 62.0) [12]	13.8 (-60.2 - 54.8)[11]	59.0 (16.0 - 81.6)[11]	32.7 (14.4 – 47.6)[13]	25.1 (14.7–34.3)[13]

(b) Vaccine effectiveness of booster doses.

Primary	Booster	Delta variant	Omicron variant
AstraZeneca	Pfizer	95.4 (95.1 - 95.6)[2]	62.4 (61.8 - 63.0)[2]
AstraZeneca	Moderna	97.0 (96.7 - 97.3)[2]	70.1 (69.5 - 70.7)[2]
AstraZeneca	AstraZeneca	82.3 (71.3 - 89.0)[2]	55.6 (44.4 - 64.6)[2]
Pfizer	Pfizer	95.1 (94.8 - 95.4)[2]	67.2 (66.5 - 67.8)[2]
Pfizer	Moderna	96.6 (96.0 - 97.1)[2]	73.9 (73.1 - 74.6)[2]
Moderna	Pfizer	94.7 (89.3 - 97.3)[2]	64.9 (62.3 - 67.3)[2]
Moderna	Moderna	96.4 (91.4 - 98.5)[2]	66.3 (63.7 - 68.8)[2]
Sinovac	Sinovac		51.0 (39.6–60.4)[13]
Janssen	Janssen		54 (43–63)[6]
Sinovac	Pfizer		63.6 (62.8 - 64.3)[14]

Table S2: Start dates of vaccination and boosters, first detection of the Delta and Omicron variants, and dates when Delta and Omicron began to dominate in the seven countries. The Delta and Omicron dominant dates in the 4th and 7th columns were reported as the first dates when the proportions of Delta and Omicron exceeded 50% in all SARS-CoV-2 viruses by genome sequencing. The eighth column was reported as the estimated diagnosis rates $\hat{\alpha}$ for the seven countries with the standard errors obtained by the bootstrap method in the parentheses.

Country	Start of vaccination	Start of Delta	Date Delta dominates	Start of booster	Start of Omicron	Date Omicron dominates	Estimated diagnosis rates
Brazil	2021-01-16	2021-05-20	2021-08-16	2021-09-19	2021-11-29	2022-01-03	0.115 (0.014)
Germany	2020-12-26	2021-03-01	2021-07-05	2021-08-29	2021-11-26	2022-01-03	0.120 (0.012)
Italy	2020-12-26	2021-04-02	2021-07-05	2021-09-19	2021-11-26	2022-01-03	0.200 (0.007)
Peru	2021-02-07	2021-06-10	2021-09-13	2021-10-14	2021-12-06	2022-01-03	0.110 (0.011)
Turkey	2021-02-11	2021-04-28	2021-06-21	2021-06-29	2021-12-06	2022-01-17	0.160 (0.011)
UK	2021-01-09	2021-02-22	2021-05-24	2021-09-29	2021-11-27	2021-12-20	0.140 (0.013)
US	2020-12-12	2021-02-23	2021-06-21	2021-08-12	2021-12-01	2021-12-20	0.100 (0.003)

Table S3: The estimated vaccine protection rates of the partial $(1-\hat{\varphi}_1)$, full $(1-\hat{\varphi}_1\hat{\varphi}_2)$ and booster vaccination $(1-\hat{\varphi}_1\hat{\varphi}_2\hat{\varphi}_3)$ against COVID-19 infection in the 6 periods considered in Figure 1.

(a) Pre-Delta period

Country	Time	Vaccine	Partial	Full
Brazil	2021-01-16 ∼ 2021-05-19	AstraZeneca, Sinovac	0.625 (0.04)	0.75 (0.03)
Germany	2020-12-26 ∼ 2021-02-28	Janssen, Moderna, AstraZeneca, Pfizer	0.56 (0.04)	0.89 (0.02)
Italy	2020-12-26 ∼ 2021-04-01	Janssen, Moderna, AstraZeneca, Pfizer	0.58 (0.04)	0.94 (0.01)
Peru	2021-02-07 ∼ 2021-06-09	AstraZeneca, Pfizer, Sinopharm	0.64 (0.04)	0.76 (0.03)
Turkey	2021-02-11 ∼ 2021-04-27	Sinovac, Pfizer	0.48 (0.04)	0.74 (0.02)
UK	2021-01-09 ∼ 2021-02-21	AstraZeneca, Pfizer	0.52 (0.04)	0.68 (0.03)
US	2020-12-12 ∼ 2021-02-22	Moderna, Pfizer	0.55 (0.04)	0.95 (0.01)
Ave (SE)			0.565 (0.02)	0.816 (0.04)

(b) Intervening I period

Country	Time	Vaccine	Partial	Full
Brazil	2021-05-20 ∼ 2021-08-15	Pfizer, AstraZeneca, Sinovac	0.52 (0.04)	0.68 (0.02)
Germany	2021-03-01 ∼ 2021-07-04	Janssen, Moderna, AstraZeneca, Pfizer	0.505 (0.04)	0.67 (0.02)
Italy	2021-04-02 ∼ 2021-07-04	Janssen, Moderna, AstraZeneca, Pfizer	0.55 (0.04)	0.7 (0.03)
Peru	2021-06-10 ∼ 2021-09-12	AstraZeneca, Pfizer, Sinopharm	0.46 (0.04)	0.73 (0.02)
Turkey	2021-04-28 ∼ 2021-06-20	Sinovac, Pfizer	0.235 (0.04)	0.49 (0.03)
UK	2021-02-22 ∼ 2021-05-23	Moderna, AstraZeneca, Pfizer	0.46 (0.04)	0.64 (0.03)
US	2021-02-23 ∼ 2021-06-20	Janssen, Moderna, Pfizer	0.675 (0.03)	0.87 (0.02)
Ave (SE)			0.486 (0.05)	0.683 (0.04)

(c) Delta-dominated period

Country	Time	Vaccine	Partial	Full
Brazil	2021-08-16 ∼ 2021-09-18	Janssen, Pfizer, AstraZeneca, Sinovac	0.385 (0.04)	0.59 (0.02)
Germany	2021-07-05 ∼ 2021-08-28	Janssen, Moderna, AstraZeneca, Pfizer	0.40 (0.03)	0.60 (0.02)
Italy	2021-07-05 ∼ 2021-09-18	Janssen, Moderna, AstraZeneca, Pfizer	0.355 (0.04)	0.57 (0.03)
Peru	2021-09-13 ∼ 2021-10-13	AstraZeneca, Pfizer, Sinopharm	0.415 (0.03)	0.61 (0.02)
UK	2021-05-24 ∼ 2021-09-28	Moderna, AstraZeneca, Pfizer	0.34 (0.03)	0.56 (0.02)
US	2021-06-21 ∼ 2021-08-11	Janssen, Moderna, Pfizer	0.48 (0.04)	0.74 (0.03)
Ave (SE)			0.396 (0.02)	0.612 (0.03)

Continued on next page

Table S3 – continued from previous page

(d) Pre-Omicron period

Country	Time	Vaccine	Partial	Full	Booster
Brazil	2021-09-19 ∼ 2021-11-28	Janssen, Pfizer, AstraZeneca, Sinovac	0.28 (0.06)	0.55 (0.02)	0.82 (0.05)
Germany	2021-08-29 ∼ 2021-11-25	Janssen, Moderna, AstraZeneca, Pfizer	0.344 (0.07)	0.59 (0.03)	0.795 (0.05)
Italy	2021-09-19 ∼ 2021-11-25	Janssen, Moderna, AstraZeneca, Pfizer	0.325 (0.06)	0.55 (0.03)	0.82 (0.06)
Peru	2021-10-14 ∼ 2021-12-05	AstraZeneca, Pfizer, Sinopharm	0.34 (0.06)	0.56 (0.03)	0.824 (0.06)
Turkey	2021-06-21 ∼ 2021-12-05	Sinovac, Pfizer	0.205 (0.05)	0.47 (0.02)	0.788 (0.6)
UK	2021-09-29 ∼ 2021-11-26	Moderna, AstraZeneca, Pfizer	0.31 (0.06)	0.54 (0.02)	0.816 (0.05)
US	2021-08-12 ∼ 2021-11-30	Janssen, Moderna, Pfizer	0.46 (0.05)	0.70 (0.03)	0.97 (0.03)
Ave (SE)			0.323 (0.03)	0.566 (0.03)	0.833 (0.02)

(e) Intervening II period

Country	Time	Vaccine	Partial	Full	Booster
Brazil	2021-11-29 ∼ 2022-01-02	Janssen, Pfizer, AstraZeneca, Sinovac	0.184 (0.04)	0.49 (0.03)	0.694 (0.07)
Germany	2021-11-26 ∼ 2022-01-02	Janssen, Moderna, AstraZeneca, Pfizer	0.30 (0.06)	0.50 (0.04)	0.70 (0.05)
Italy	2021-11-26 ∼ 2022-01-02	Janssen, Moderna, AstraZeneca, Pfizer	0.295 (0.04)	0.53 (0.03)	0.718 (0.05)
Peru	2021-12-06 ∼ 2022-01-02	AstraZeneca, Pfizer, Sinopharm	0.265 (0.05)	0.51 (0.05)	0.706 (0.06)
Turkey	2021-12-06 ∼ 2022-01-16	Sinovac, Pfizer	0.055 (0.06)	0.37 (0.05)	0.622 (0.06)
UK	2021-11-27 ∼ 2022-12-19	Moderna, AstraZeneca, Pfizer	0.116 (0.05)	0.48 (0.05)	0.688 (0.07)
US	2021-12-01 ∼ 2022-12-19	Janssen, Moderna, Pfizer	0.34 (0.04)	0.56 (0.04)	0.736 (0.04)
Ave (SE)			0.222 (0.04)	0.491 (0.02)	0.695 (0.01)

(f) Omicron-dominated period

Country	Time	Vaccine	Partial	Full	Booster
Brazil	2022-01-03 ∼ 2022-03-15	Janssen, Pfizer, AstraZeneca, Sinovac	0.07 (0.04)	0.38 (0.03)	0.628 (0.06)
Germany	2022-01-03 ∼ 2022-03-15	Janssen, Moderna, AstraZeneca, Pfizer	0.115 (0.04)	0.41 (0.05)	0.646 (0.06)
Italy	2022-01-03 ∼ 2022-03-15	Janssen, Moderna, AstraZeneca, Pfizer, Novavax	0.16 (0.05)	0.44 (0.04)	0.664 (0.07)
Peru	2022-01-03 ∼ 2022-03-15	AstraZeneca, Pfizer, Sinopharm	0.10 (0.04)	0.40 (0.03)	0.64 (0.06)
Turkey	2022-01-17 ∼ 2022-03-15	Sinovac, Pfizer, Turkovac	0.038 (0.07)	0.26 (0.04)	0.556 (0.06)
UK	2021-12-20 ∼ 2022-03-15	Moderna, AstraZeneca, Pfizer	0.04 (0.06)	0.36 (0.02)	0.616 (0.06)
US	2021-12-20 ∼ 2022-03-15	Janssen, Moderna, Pfizer	0.285 (0.06)	0.45 (0.04)	0.67 (0.07)
Ave (SE)			0.115 (0.03)	0.386 (0.02)	0.631 (0.01)

Table S4: The observed and the projected numbers (in thousands), and the percentages (%) of confirmed cases (a) and deaths (b) under the two scenarios (with no and the only-partial vaccination) relative to the observed numbers during the period from the start of partial vaccination to start of booster vaccination. The 95% confidence intervals are attached to the projected numbers and percentages of the two scenarios.

(a) Confirmed cases

Country	Observed	Scenarios			
		No vaccination		Partial vaccination	
		Cases	Percentage	Cases	Percentage
Brazil	12762	22019 (18344, 25695)	173 (144, 201)	14507 (13358, 15656)	114 (105, 123)
Germany	2325	4000 (3401, 4600)	172 (146, 198)	2649 (2383, 2915)	114 (102, 125)
Italy	2597	6980 (6413, 7546)	269 (247, 291)	3369 (3249, 3488)	130 (125, 134)
Peru	1006	1210 (1064, 1355)	120 (106, 135)	1070 (956, 1184)	106 (95, 118)
Turkey	2856	4075 (3809, 4341)	143 (133, 152)	3225 (3031, 3418)	113 (106, 120)
UK	4796	23262 (21753, 24771)	485 (454, 516)	14606 (13156, 16057)	305 (274, 335)
US	20341	98055 (88040, 108070)	482 (433, 531)	46954 (40654, 53253)	231 (200, 262)
Total	46683	159601 (142825, 176378)	342 (306, 378)	86380 (76788, 95972)	185 (164, 206)

(b) Deaths

Country	Observed	Scenarios			
		No vaccination		Partial vaccination	
		Deaths	Percentage	Deaths	Percentage
Brazil	382	582 (494, 670)	152 (129, 176)	418 (385, 452)	110 (101, 118)
Germany	63	72 (65, 80)	116 (104, 127)	64 (58, 69)	102 (93, 111)
Italy	59	91 (87, 96)	155 (148, 163)	63 (61, 65)	108 (104, 111)
Peru	92	101 (89, 112)	110 (98, 122)	95 (85, 104)	103 (93, 114)
Turkey	23	32 (30, 34)	140 (132, 149)	25 (24, 27)	112 (105, 119)
UK	55	108 (101, 115)	195 (182, 207)	83 (76, 90)	150 (137, 162)
US	317	829 (754, 904)	262 (238, 285)	460 (425, 495)	145 (134, 156)
Total	990	1815 (1620, 2010)	183 (164, 203)	1208 (1114, 1302)	122 (113, 132)

Table S5: The observed and the projected numbers (in thousands), and the percentages (%) of confirmed cases (a) and deaths (b) under the three scenarios (with no booster, half and double the actual booster vaccination rates) relative to the observed numbers during the period from the start of booster vaccination to March 15, 2022. The 95% confidence intervals are attached to the projected numbers and percentages of the three scenarios.

(a) Confirmed cases

Country	Observed	Scenarios					
		No booster		Half booster		Double booster	
		Cases	Percentage	Cases	Percentage	Cases	Percentage
Brazil	8239	11598 (10207, 12989)	141 (124, 158)	9887 (8849, 10926)	120 (107, 133)	6528 (5751, 7306)	79 (70, 89)
Germany	13668	18589 (16773, 20404)	136 (123, 149)	15369 (14274, 16463)	112 (104, 120)	8322 (7688, 8956)	61 (56, 66)
Italy	8913	12452 (11990, 12914)	140 (135, 145)	10124 (9765, 10483)	114 (110, 118)	5583 (5014, 6152)	63 (56, 69)
Peru	1349	1798 (1398, 2197)	133 (104, 163)	1591 (1317, 1865)	118 (98, 138)	1136 (950, 1322)	84 (70, 98)
Turkey	9160	14961 (12312, 17610)	163 (134, 192)	11857 (10639, 13075)	129 (116, 143)	5852 (4617, 7086)	64 (50, 77)
UK	12113	15692 (14779, 16605)	130 (122, 137)	12588 (12077, 13098)	104 (100, 108)	5851 (5266, 6436)	48 (43, 53)
US	43056	56268 (52582, 59955)	131 (122, 139)	49670 (47601, 51738)	115 (111, 120)	35548 (33089, 38006)	83 (77, 88)
Total	96498	131358 (120041, 142675)	136 (124, 148)	111085 (104522, 117648)	115 (108, 122)	68820 (62374, 75265)	71 (65, 78)

(b) Deaths

Country	Observed	Scenarios					
		No booster		Half booster		Double booster	
		Deaths	Percentage	Deaths	Percentage	Deaths	Percentage
Brazil	65	82 (73, 92)	127 (113, 142)	74 (66, 82)	114 (102, 126)	57 (51, 62)	87 (78, 96)
Germany	34	40 (35, 45)	120 (105, 135)	36 (33, 39)	105 (96, 115)	27 (25, 29)	79 (73, 85)
Italy	27	36 (34, 38)	134 (125, 142)	30 (28, 31)	111 (105, 116)	18 (16, 20)	66 (58, 74)
Peru	12	15 (12, 18)	127 (102, 153)	14 (11, 16)	115 (95, 135)	10 (9, 12)	87 (75, 99)
Turkey	47	72 (60, 84)	153 (128, 178)	59 (53, 64)	125 (113, 136)	33 (27, 38)	70 (58, 81)
UK	27	33 (31, 35)	124 (116, 132)	27 (25, 29)	101 (95, 107)	15 (14, 16)	55 (51, 60)
US	350	425 (404, 447)	122 (115, 128)	388 (373, 404)	111 (106, 115)	308 (294, 322)	88 (84, 92)
Total	561	704 (649, 759)	126 (116, 135)	627 (590, 664)	112 (105, 118)	467 (435, 499)	83 (78, 89)

Table S6: The peak (maximum) values of the observed and the projected numbers (in millions) of active confirmed cases during the post-vaccine period with the days that the projected numbers of active confirmed cases under each scenario would exceed the observed peak. The percentages (%) of the projected peak values over the observed peak values are provided in parentheses. The five scenarios are (i) no vaccination at all; (ii) only partial vaccination; (iii) no booster, (iv) half and (v) twice the booster up-take.

Country	Observed	Scenarios									
		No vaccination		Partial vaccination		No booster		Half booster		Double booster	
	Peak	Peak	Days	Peak	Days	Peak	Days	Peak	Days	Peak	Days
Brazil	1.89	11.82 (624)	76	12.07 (637)	71	2.62 (138)	36	2.19 (116)	24	1.31 (69)	0
Germany	2.53	6.35 (251)	71	6.27 (248)	65	3.53 (140)	41	2.94 (116)	27	1.38 (54)	0
Italy	1.97	3.44 (175)	70	5.83 (296)	66	2.86 (145)	36	2.23 (113)	21	1.1 (56)	0
Peru	0.41	3.85 (940)	79	2.86 (697)	71	0.51 (123)	23	0.44 (108)	14	0.3 (74)	0
Turkey	1.20	2.77 (231)	111	2.11 (176)	156	2.12 (178)	59	1.6 (134)	42	0.66 (55)	0
UK	1.76	4.04 (230)	80	2.13 (121)	76	2.43 (138)	51	1.74 (99)	0	0.65 (37)	0
US	8.29	31.89 (385)	76	20.94 (253)	72	11.2 (135)	31	9.45 (114)	21	5.68 (69)	0

Table S7: The estimated vaccine protection rates of the partial, full and booster vaccinated against COVID-19 infection in the intervening II and Omicron-dominated periods in the sensitivity analyses of the symptomatic rate and the average time duration from recovery to loss of natural immunity. Compared to the results in Table S3 (e) and (f), the largest difference between VPRs in the main analysis and those in sensitivity analysis of asymptomatic rate was 8.6% for the partial vaccination in the Omicron-dominated period in Italy, for other vaccinations, periods and countries the differences were no more than 3.4%, and the average of the absolute differences was 1.26% (SE: 0.23%). And the largest difference between VPRs in the main analysis and those in the sensitivity analysis of duration of natural immunity was 5% for the full vaccination in the Intervening II period in Turkey, for other vaccinations, periods and countries the differences were no more than 3%, and the average of the absolute differences was 0.94% (SE: 0.17%).

Country	Asymptomatic rate						Time from recovery to loss of natural immunity					
	Intervening II			Omicron-dominated			Intervening II			Omicron-dominated		
	Partial	Full	Booster	Partial	Full	Booster	Partial	Full	Booster	Partial	Full	Booster
Brazil	0.167	0.51	0.706	0.07	0.38	0.628	0.20	0.50	0.70	0.085	0.39	0.634
Germany	0.28	0.52	0.712	0.115	0.41	0.646	0.28	0.52	0.712	0.10	0.40	0.64
Italy	0.28	0.52	0.712	0.246	0.42	0.652	0.295	0.53	0.718	0.13	0.42	0.652
Peru	0.235	0.49	0.694	0.10	0.40	0.64	0.265	0.51	0.706	0.07	0.38	0.628
Turkey	0.085	0.39	0.634	0.051	0.27	0.562	0.072	0.42	0.594	0.038	0.26	0.556
UK	0.15	0.50	0.70	0.055	0.37	0.622	0.116	0.48	0.688	0.04	0.36	0.616
US	0.34	0.56	0.736	0.298	0.46	0.676	0.34	0.56	0.736	0.298	0.46	0.676
Average	0.220	0.499	0.699	0.134	0.387	0.632	0.224	0.503	0.693	0.109	0.381	0.629

References

[1] F. P. Polack, S. J. Thomas, N. Kitchin, *et al.*, "Safety and efficacy of the BNT162b2 mRNA Covid-19 vaccine," *New England Journal of Medicine*, vol. 383, no. 27, pp. 2603–2615, 2020. DOI: 10.1056/NEJMoa2034577.

[2] N. Andrews, J. Stowe, F. Kirsebom, *et al.*, "Covid-19 vaccine effectiveness against the omicron (b.1.1.529) variant," *New England Journal of Medicine*, vol. 386, no. 16, pp. 1532–1546, 2022. DOI: 10.1056/NEJMoa2119451.

[3] L. R. Baden, H. M. El Sahly, B. Essink, *et al.*, "Efficacy and safety of the mRNA-1273 SARS-CoV-2 vaccine," *New England Journal of Medicine*, vol. 384, no. 5, pp. 403–416, 2021. DOI: 10.1056/NEJMoa2035389.

[4] J. Sadoff, G. Gray, A. Vandebosch, *et al.*, "Safety and efficacy of single-dose Ad26.COV2.S vaccine against Covid-19," *New England Journal of Medicine*, vol. 384, no. 23, pp. 2187–2201, 2021. DOI: 10.1056/NEJMoa2101544.

[5] NBC Boston, *Pfizer, Moderna, J&J vaccines: Efficacy as delta variant concerns rise*, https://www.nbcboston.com/news/local/pfizer-moderna-jj-vaccines-efficacy-as-delta-variant-concerns-rise/2419162/, 2021.

[6] K. Natarajan, N. Prasad, K. Dascomb, *et al.*, "Effectiveness of homologous and heterologous covid-19 booster doses following 1 ad.26.cov2.s (janssen [johnson & johnson]) vaccine dose against covid-19–associated emergency department and urgent care encounters and hospitalizations among adults — vision network, 10 states, december 2021–march 2022," *MMWR. Morbidity and Mortality Weekly Report*, vol. 71, pp. 495–502, Apr. 2022. DOI: 10.15585/mmwr.mm7113e2.

[7] M. Voysey, S. A. C. Clemens, S. A. Madhi, *et al.*, "Safety and efficacy of the ChAdOx1 nCoV-19 vaccine (AZD1222) against SARS-CoV-2: An interim analysis of four randomised controlled trials in Brazil, South Africa, and the UK," *The Lancet*, vol. 397, no. 10269, pp. 99–111, 2021.

[8] M. Voysey, S. A. Costa Clemens, S. A. Madhi, *et al.*, "Single-dose administration and the influence of the timing of the booster dose on immunogenicity and efficacy of ChAdOx1 nCoV-19 (AZD1222) vaccine: A pooled analysis of four randomised trials," *The Lancet*, vol. 397, no. 10277, pp. 881–891, 2021. DOI: 10.1016/S0140-6736(21)00432-3.

[9] J. L. Bernal, N. Andrews, C. Gower, *et al.*, "Effectiveness of Covid-19 vaccines against the B.1.617.2 (Delta) variant," *New England Journal of Medicine*, vol. 385, no. 7, pp. 585–594, 2021. DOI: 10.1056/NEJMoa2108891.

[10] N. Al Kaabi, Y. Zhang, S. Xia, *et al.*, "Effect of 2 inactivated SARS-CoV-2 vaccines on symptomatic COVID-19 infection in adults: A randomized clinical trial," *JAMA*, vol. 326, no. 1, pp. 35–45, 2021, ISSN: 0098-7484. DOI: 10.1001/jama.2021.8565.

[11] X. Li, Y. Huang, W. Wang, *et al.*, "Effectiveness of inactivated SARS-CoV-2 vaccines against the Delta variant infection in Guangzhou: A test-negative case–control real-world study," *Emerging Microbes & Infections*, vol. 10, no. 1, pp. 1751–1759, 2021. DOI: 10.1080/22221751.2021.1969291.

[12] R. Palacios, A. Batista, C. Albuquerque, *et al.*, "Efficacy and safety of a COVID-19 inactivated vaccine in healthcare professionals in Brazil: The PROFISCOV study," *SSRN Electronic Journal*, 2021. DOI: 10.2139/ssrn.3822780.

[13] M. E. McMenamin, J. Nealon, Y. Lin, *et al.*, "Vaccine effectiveness of one, two, and three doses of bnt162b2 and coronavac against covid-19 in hong kong: A population-based observational study," *The Lancet Infectious Diseases*, 2022, ISSN: 1473-3099. DOI: https://doi.org/10.1016/S1473-3099(22)00345-0.

[14] T. Cerqueira-Silva, V. de Araujo Oliveira, E. S. Paixão, *et al.*, "Duration of protection of CoronaVac plus heterologous BNT162b2 booster in the Omicron period in Brazil," *Nature Communications*, vol. 13, no. 1, p. 4154, 2022. DOI: 10.1038/s41467-022-31839-7.
